# Phylogenies from dynamic networks

**DOI:** 10.1371/journal.pcbi.1006761

**Published:** 2019-02-26

**Authors:** Cornelia Metzig, Oliver Ratmann, Daniela Bezemer, Caroline Colijn

**Affiliations:** 1 Dept of Electronic Engineering and Computer Science, Queen Mary University of London, London, United Kingdom; 2 Dept of Mathematics, Imperial College London, London, United Kingdom; 3 Stichting HIV Monitoring, Amsterdam, the Netherlands; 4 Dept of Mathematics, Simon Fraser University, Burnaby, Canada; University of California Irvine, UNITED STATES

## Abstract

The relationship between the underlying contact network over which a pathogen spreads and the pathogen phylogenetic trees that are obtained presents an opportunity to use sequence data to learn about contact networks that are difficult to study empirically. However, this relationship is not explicitly known and is usually studied in simulations, often with the simplifying assumption that the contact network is static in time, though human contact networks are dynamic. We simulate pathogen phylogenetic trees on dynamic Erdős-Renyi random networks and on two dynamic networks with skewed degree distribution, of which one is additionally clustered. We use tree shape features to explore how adding dynamics changes the relationships between the overall network structure and phylogenies. Our tree features include the number of small substructures (cherries, pitchforks) in the trees, measures of tree imbalance (Sackin index, Colless index), features derived from network science (diameter, closeness), as well as features using the internal branch lengths from the tip to the root. Using principal component analysis we find that the network dynamics influence the shapes of phylogenies, as does the network type. We also compare dynamic and time-integrated static networks. We find, in particular, that static network models like the widely used Barabasi-Albert model can be poor approximations for dynamic networks. We explore the effects of mis-specifying the network on the performance of classifiers trained identify the transmission rate (using supervised learning methods). We find that both mis-specification of the underlying network and its parameters (mean degree, turnover rate) have a strong adverse effect on the ability to estimate the transmission parameter. We illustrate these results by classifying HIV trees with a classifier that we trained on simulated trees from different networks, infection rates and turnover rates. Our results point to the importance of correctly estimating and modelling contact networks with dynamics when using phylodynamic tools to estimate epidemiological parameters.

## Author summary

Understanding whether and how transmission patterns are revealed by branching patterns in phylogenetic trees for pathogens remains a challenging research question. Besides the diversification of the pathogen, branching patterns depend strongly on the host contact structure as it shapes opportunities for the pathogen to reproduce. However, the host contact network is often difficult to study, in particular as it evolves in time. In this paper we perform a simulation study on three different dynamic networks, on which we simulate transmission trees. We use a simple Erdős-Renyi random network and two more realistic networks with skewed degree distribution, where one is also clustered. We convert transmission trees into phylogenetic trees and analyze them with different tree statistics like imbalance measures, counts of small substructures, and measures containing the branch lengths. We study the tree features with principal component analysis and with supervised learning methods, and find that network dynamics and network type can strongly influence the shape of phylogenetic trees. This implies that using phylogenetic trees from a mis-specified network type and dynamic can lead to poor phylodynamic estimation of transmission parameters. We illustrate this with HIV phylogenetic trees constructed from viral sequences of patients in the Dutch ATHENA cohort, and from sequences of the Los Alamos Sequence database.

## Introduction

Understanding whether and how the transmission patterns of a pathogen are revealed by branching patterns in pathogen phylogenetic trees remains a challenging research question. Alongside the stochastic diversification of the pathogen on the short time scales of an infectious disease outbreak, branching patterns in the pathogen’s phylogenetic tree also depend strongly on the underlying transmission pattern [1] and the host contact structure, as these shape the pathogen’s reproductive opportunities.

The role of networks in epidemic spreading has been studied extensively in past decades [2–12]. The topology of the host contact network plays a crucial role in setting the epidemic threshold, the epidemic size and the most effective interventions. Network properties also play a role in determining which individuals are at high risk of infection. Naturally, modellers seek to inform simulated networks with individual-level data from real populations. Respondent-driven sampling [13, 14], snowball sampling or questionnaires [15] are several approaches to gathering these data, but all are challenging: people do not always remember how many people they have been in contact with, and in some contexts (such as injection drug use or sexual behaviour), contact is stigmatized or even illegal. As a result, individuals may not wish to report contacts to public health practitioners.

Recently there has been interest in using genetic data from pathogens, together with phylogenetic and phylodynamic tools, to estimate the parameters of human contact networks [16–19]. This is appealing, in that data now accessible with high-throughput sequencing technologies (pathogen sequences, at a level of resolution that makes detecting even small amounts of genetic variation feasible) can reveal information about a fundamental population-level structure (the network). Sequences can show patterns of descent, and pathogens transmitted directly from human to human need human contact networks to have descendants. Since networks are difficult to observe directly and phylogenetic trees in principle contain some information about them, researchers have used a variety of tools to relate pathogen phylogenetic trees to the underlying contact network’s degree distribution, connectivity and clustering [17, 20]. This method has been of particular interest for HIV phylogenies [21–24].

Studies have reported varying strengths of the effect of the contact network on the phylogeny. For example, [25] found a very weak influence of the network’s clustering coefficient when the degree distribution is held constant, [26] studied the shapes of phylogenies from simulated genetic data and found a moderate influence of the underlying network degree distribution, though “clustering” in phylogenetic trees did not parallel the heterogeneity in the degree distribution, and network dynamics shape phylogenies as well. [21] found a relatively stong effect of the variance in degree distribution and of the average pathlength of the network on the shapes of phylogenies. Also, within-host viral diversity affects the link between network structures and phylogenies [23], as do the basic reproduction number and other details of the process [27, 28]. It is therefore reasonable to assume that details of timing of infection, in-host selection, selection at the population level and other factors may also affect the relationship between contact networks and phylogies.

Human contact networks are self-organizing systems with certain general characteristics; one approach to modelling human host networks is to perform simulations that are able to reproduce those characteristics. Key characteristics include a short average pathlength (small-world property) [29], clustering [30] and a scale-free (or at least highly skewed) degree distribution [31, 32]. In particular, networks with a skewed degree distribution have received much attention for epidemic spreading, as they yield significantly different transmission patterns from a homogeneously mixed population. Depending on the transmission pathway, there is evidence that networks can have an exponential degree distribution [13, 33] or a scale-free degree distribution, found in various social networks [34–36], and in human contact networks [37–39]. The Barabasi-Albert model [40] in particular is a much-studied process by which scale-free degree distributions may emerge. It is based on the idea of preferential attachment: nodes attach preferentially to existing nodes that already have many links.

Preferential attachment is a plausible rationale for many applications (fame, publicity). It describes a constantly growing network, or a static network if the growth is halted. In contrast, human host contact networks are often dynamic, but may not be growing in size over time. Instead, they have population turnover [5, 41], with individuals entering and leaving a network as time goes on. Especially for chronic infections like TB, HCV or HIV [42], people may enter and exit the network over shorter timescales than the length of the infectious period. The number of contacts that individuals accumulate over time is significantly larger than the number of contacts at one point in time.

Furthermore, many of the observations underlying reports of scale-free degree distributions in human contact networks are derived from reports of the *cumulative* numbers of contacts that individuals have over a long period (for example over one year [32, 43], or accumulated to date). Accordingly, it may not be appropriate to compare simulated transmission dynamics in models where individuals’ degrees are modelled from observed *accumulated* numbers of contacts to transmission where degrees are taken as the *instantaneous* (or even shorter-term) numbers of contacts. The static network (with degrees modelled on data for the number of contacts accumulated over long time periods) can be a very poor approximation of the true dynamic network; outbreaks can spread faster in such a static network due to the potentially very high numbers of simultaneous contacts.

In using phylodynamic tools to estimate network parameters from pathogen phylogenies, it is typically assumed that the contact network is static in time; one seeks network parameters that produce pathogen phylogenetic trees that are similar to observed trees, conditional on the static network assumption (and perhaps also on assumptions about the degree distribution, clustering patterns and other network attributes). Whatever the details, inferred quantities such as degree distribution, the average number of partners and the infection rate are influenced by assumptions about the network, including the static assumption.

The duration of infectiousness and the time scale of the network dynamics must affect the relationship between pathogen phylogenies and network parameters. Clearly, no individual has thousands of contacts over a week; reports of degrees that are orders of magnitude higher than the average are from data aggregated over long time periods; where an infectious duration is of the order of weeks or a few months, the scale-free property is unlikely to hold. These issues are presented briefly in [26] and [44, 45].

In this paper, we investigate the effect of human host network dynamics on pathogen phylogenies. Our study focuses on simulations, and on the relationship between network assumptions and estimates of transmission parameters. We compare simulated phylogenies from outbreaks on static and dynamic networks, and we explore the effect of the turnover rate at which individuals enter and leave the system. We also study the effect of the network characteristics on the phylogenies. For this, we use networks with binomial degree distribution and skewed degree distribution, as well as clustered and unclustered networks. We explore the effect of the infection rate and the mean number of contacts. We study how the features of the underlying networks affect phylogenetic trees with various tree statistics. Finally, we turn to phylodynamic inference of HIV transmission parameters and illustrate our main results using HIV sequence data from the Dutch ATHENA cohort and Los Alamos. In particular, we characterise the impact of alternative assumptions on human contact network dynamics on estimation of key transmission parameters including R0.

## Methods

We simulate the human contact network with the algorithms described in section. First, we allow the networks to converge to a stationary state in terms of degree distribution; in this stationary state, networks are still dynamic in the sense that people enter and exit. Then, an outbreak is simulated on the networks while they continue to evolve. One person is infected and, with a constant infection rate per contact, the infection can spread. The resulting infection trees are converted into a phylogenetic trees (see section). Unlike the Barabasi-Albert (BA) model, our approach allows a skewed degree distribution to emerge while keeping the size and total degree fixed. Throughout, individuals enter and leave the network and links are formed and dissolved. In contrast, in the BA model, nodes and links are continuously added and remain in the network. We set a constant number of tips in our trees. We use tree shape and length statistics, detailed in section, to compare phylogenetic trees.

### Network algorithms

We use an algorithm for a “skewed-clustered” network which generates a network with a skewed degree distribution and positive transitivity [38]. To understand what these features add, we also use skewed (but not particularly clustered) networks, and an Erdős-Renyi random network. These all have a stationary average number of contacts and stationary degree distribution, while people are entering and exiting the network. This entry and exit happens with a turnover rate *δ*, which is the ratio between the number of people entering per time step to the number of people in the network. Networks are simulated in discrete time. In each time step the following steps happen:

#### Random network

A person enters the network and gets connected to a person chosen at random. Further links are added between randomly chosen people in the network to keep the average degree constant. People exit the system at the given rate. When a person leaves the network, their links are broken. The degree distribution in this algorithm converges to a binomial degree distribution.

#### Skewed network

A person enters the network and forms a partnership (link) with one other person *j*, where the probability to select someone as partner is proportional to that person’s current number of partnerships (degree). To maintain a constant number of links despite the fact that individuals leave the system, additional links are introduced. For this, a first person *i* is picked with probability proportional to its number of contacts. Node *i* is then linked to a second person *j*, who is again picked with probability proportional to person *j*’s number of contacts. People exit the system at a given rate, and their links are all broken. If a person is left without any links because their partners have left the network, they are connected to existing nodes, again with probability proportional to a node’s degree.

It can be shown theoretically [46] that the degree distribution in this process converges to a power law degree distribution with an exponential cutoff; the cutoff strength increases with decreasing number of people (nodes), and also is increased when the mean degree is decreased. For mean degree ≈ 3 and a network size of 1000 nodes, the cutoff is so strong that the degree distribution can always be approximated by an exponential distribution.

#### Skewed-clustered network

This is a variant of the algorithm described above. Again a person *i* enters the network, and another person *j* to receive an additional link is picked, with probability proportional to its current degree. Additional links are added, where the first neighbour is again picked with probability proportional to its number of contacts. The second neighbour is picked

(i) among neighbours of second degree (neighbours’ neighbours) (at random)
(ii) if that is not possible, among neighbours of third degree (at random)
(iii) if that is not possible, in the whole network, with probability proportional to a node’s degree.

After people exit at a given rate, those left without neighbours are connected to existing nodes, with probability proportional to a person’s degree, and other links are broken.

**Fig 1:**
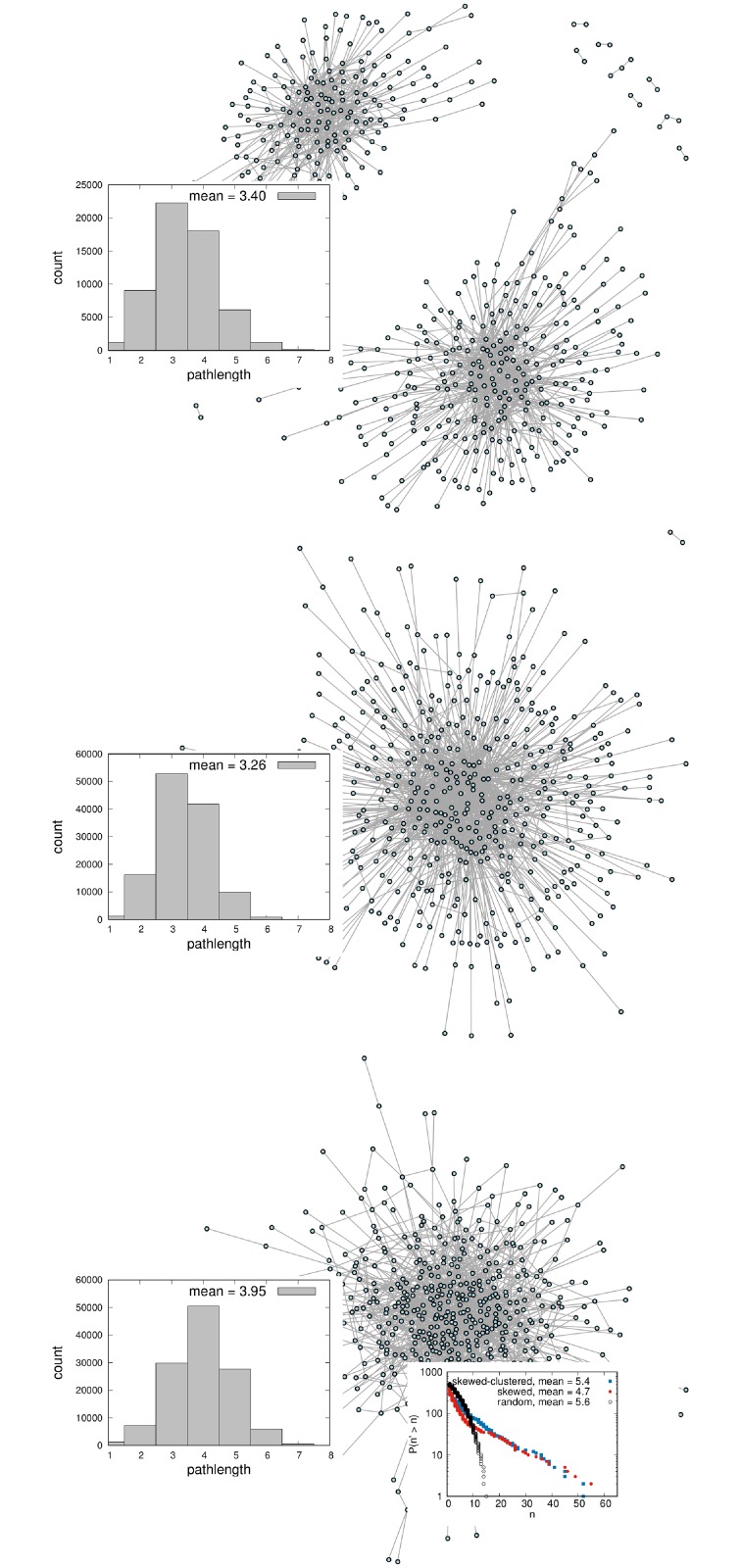
Topologies of a dynamic skewed-clustered network (top), a dynamic skewed network (middle) and a dynamic random network (bottom) at one point in time. Turnover rate (probability to leave the network in one timestep) *δ* = 0.1. The inlays on the left show the pathlength distribution. The skewed network has a much shorter pathlength than the random network (for same mean degree), and skewed-clustered network has slightly longer average pathlength, but still shorter than the random network. This relationship holds for a wide range of mean degrees. The inlay on the right shows the counter-cumulative degree distribution in loglinear scale, which are power laws with exponential cutoff for the skewed-clustered network and skewed network (blue and red), and binomial for the random network (black).

The stationary state of the degree distribution is again a power law with exponential cutoff, with a higher decay constant as in the skewed network (for low mean degree *d* < 3). At a given point in time, not all nodes in the network are necessarily connected to one component (see Fig 1). The clustering coefficient, or transitivity, is defined as the ratio of the number of triangles to the number of connected triplets [29]. Rules (i) and (ii) cause the transitivity to be higher than it is in the the skewed network (for all system sizes, here the transitivity is ≈ 0.15).

#### Time-integrated networks

We also compute time-integrated networks, i.e we let the networks evolve with entry and exit, and create (unweighted) networks of all nodes that have *ever* been in the network, where two nodes are connected if there has *ever* been a link between them. As a consequence, the time-integrated network has many more links than a dynamic network at a specific point in time. The degree distribution of the time-integrated networks has a higher mean and more than the degree distribution of the instantaneous networks. Both dynamic and time-integrated static networks have the same answer to the question “How many contacts have you had in a given time?”, so they can be modelled using the same source of input data (e.g. questionnaires [15]).

### Outbreak simulation

In our simulations, we begin with one infected individual who then infects neighbours at a constant infection rate per contact, after which the neighbours can infect their respective neighbours in the next time step, and so forth. Infected individuals stay infected throughout the simulation, modelling a long-term infection. This simulates an outbreak on these dynamic networks. There is at least one time step between an individual becoming infected and infecting a neighbour, and we model a positive time between any two infection events by adding a small positive time to the infection events of one iteration, such that they occur with equal time lapses.

We extract what would be the “true timed phylogeny” of the pathogen given the transmission tree in our network, under the assumption that hosts carry a single pathogen lineage. To do this we form a binary branching tree in which each host corresponds to a tip in the phylogeny and branch lengths correspond to time. Since we know the true transmission tree and its timing, this can be done by tracking the infectors, infectees and the time between infection events. This is available in the getLabGenealogy function in the R package PhyloTop [47]. The simulation of the outbreak is stopped after a time such that the phylogenetic trees all have the same number of tips.

### Topological summary measures of trees

We compute features of the phylogenies with software sources listed in Table 1.

1. **Number of substructures**
  **Cherries**: Substructure consisting of two tip descendants
  **Pitchforks**: Substructure consisting of three tips
2. **Imbalance measures**
  **Sackin Index**: Average number of internal nodes *N*_*i*_ between each tip *i* and the root of the phylogenetic tree $S^{n}=\frac{1}{n}\sum_{i=1}^{n}N_{i}$, [48, 49]
  **Colless Index**: It compares the number of tips that descend on the left and right (*L* and *R*) from each internal node, and averages over these differences |*L* − *R*| [49, 50].
3. **Other tree measures**
  **Maximum Height**: Maximum height of tips in the tree.
  **Average Size of Ladder**: Ladder structures [1] consisting of a connected set of internal nodes with a single tip descendant
  **IL numbers**: Number of internal nodes with a single tip child.
4. **Centrality measures and general network measures**
  **Maximum Betweenness**: Maximum number of shortest paths that pass through a particular node.
  **Wiener Index**: Sum of the lengths of the shortest paths between all pairs of nodes.
  **Maximum Closeness**: Sum of lengths of the shortest paths between one node and all other nodes (maximum thereof).
  **Average Pathlength**: Average distance between two nodes.
  **Diameter**: Longest possible path between two nodes in the tree.
5. **Tree measures that use the edge length**
  **Branching next index (BNI)**: We compare the extent to which a node that branches at time *t* is chronologically next to branch; in other words, does branching now make it more or less likely that a node will branch next? If a node’s child is chronologically next to branch following the node itself, we say the node has the ‘branching next’ property (*s*_*i*_ = 1). We add and rescale the sum of *s*_*i*_ over all internal nodes *i* in the tree (except the root and the last node to branch). *s*_*i*_ is a Bernoulli random variable whose expected value is *p*_*i*_ = 2/*k*_*i*_, where *k*_*i*_ is the number of lineages in the tree that exist at time *t*_*i*_ + *ϵ*, in the limit as *ϵ* → 0, where *t*_*i*_ is the time of node *i* and *ϵ* > 0. We define the BNI as $\frac{\sum_{i}s_{i}-p_{i}}{\sqrt{\sum_{i}p_{i}\left(1-p_{i}\right)}}$
  **Generalised branching next (MNI)**: Extending the BNI concept, we ask whether one of the next *m* branching events (chronologically) in the tree descends from the current node, in which case we set *d*_*i*_ = 1 for node *i*. We sum and rescale *d*_*i*_, as with *s*_*i*_, over the tree to create this summary statistic. We let *k*_*ij*_, *j* = 1, …, *m* be the numbers of lineages immediately after the *j*′*th* branching event following node *i* (in the entire tree). We define *q*_*i*_ = ∏_*j*_(1 − 1/*k*_*ij*_) and normalise by setting MNI to $\frac{\sum_{i}d_{i}-q_{i}}{\sqrt{\sum_{i}q_{i}\left(1-q_{i}\right)}}$. Since now they are not independent we use every *m*′th node *i* rather than every node.
  **Length statistics** We use the mean of the path length from the internal nodes of the tree to its root, as well as the median, variance, skewness and kurtosis of this set of path lengths.

**Table 1:** References of tree features.

Cherries	[47]
Pitchforks	[47]
Sackin Index	[47]
Colless Index	[47]
Maximum Heigh	[47]
Average Size of Ladder	[47]
IL numbers	[47]
Maximum Betweenness	treeCentrality [51]
Wiener Index	treeCentrality [51]
Maximum Closeness	treeCentrality [51]
Average Pathlength	treeCentrality [51]
Diameter	treeCentrality [51]
Branching next index	phyloTop
Generalised branching next	phyloTop
Mean of length from internal nodes to root	(own)

### Analysis approach

We use two approaches to understand how the underlying contact network affects the tree features. The first is to visualise the results using principal components analysis (PCA) on the matrix of features described above. The matrix values are scaled such that the mean is zero, and normalized such that variance is 1, as is standard in PCA. This visually illustrates the extent to which these features discriminate between phylogenetic trees derived from different contact networks. However, visual separation on a 2-dimensional PCA plot is a limited measure of how informative the features are of the contact network. Thus, we also explore this quantitatively using both K-nearest neighbours and random forest classification. We attempt to classify the network (random, skewed or skewed-clustered) based on the features. We assess accuracy in these binary and categorical classifications when the underlying network model correct, and when it is mis-specified. We also attempt to classify the transmission rate. For this goal we use trees from simulated outbreaks where we distributed the transmission rate *β* uniformly. We grouped these trees into bins depending on the underlying *β* and train classifiers on the tree features with the aim of predicting the bin of *β* for a test set. We study a scenario where turnover rate *δ* and mean degree $\hat{d}$ are distributed uniformly, and a scenario where they are kept constant.

### Application to HIV

Partial nucleotide HIV-1 polymerase sequences were obtained as described previously from patients in the ATHENA national observational HIV cohort in the Netherlands (by June 2015) [52]. We used the first sequence per patient, with a minimum of 750 nucleotides length. No patient information was included in the analysis. Sequences were aligned with Clustal Omega 1.1.0 [53] and manually checked and adjusted. HIV-1 subtyping was performed with COMET v1.3 [54] and 6912 subtype B sequences were considered for further analysis. In addition we retrieved 19,459 HIV-1 subtype B sequences from the Los Alamos database (by September 2017) [55], with a minimum length of 1000 nucleotides overlap to the ATHENA alignment. Excluding sites with less than 75% coverage, and with IAS resistant mutations 2015 removed This resulted in a sequence alignment of 1,128 nucleotides length [56]). Viral phylogenies were reconstructed with FastTree version 2 [57].

From this tree we identify 90 non-intersecting clades in the specified size range 100-151, using a depth first search approach. The mean number of tips in the clades was 127. 86 out of 90 clades contained samples from the ATHENA cohort, with a fraction between 0.01 and 0.97. Overall, the clades we extracted contained 8326 sequences from the Los Alamos data and 3186 from the Dutch HIV-1 ATHENA cohort. We compared the HIV clades with simulated trees from different networks and to trees simulated on the same network, but with varying infection rates. We trained random forest and K-nearest neighbour classifiers on tree features from the simulated networks, and used the features from the HIV clades as a test set. The simulated trees (the training set) had 100 tips. We then used the classifiers to predict the network type or infection rate for the HIV clades.

### Overview of scenarios

We used principal component analysis to study different types of networks, different mean degree and infection rate for a given network, as well as different turnover rates and a time-integrated static network (see all scenarios in Table 2). We also trained classifiers on the networks in order to predict infection rate, turnover rate and network type (see all scenarios in Table 3).

**Table 2:** Summary of the simulation scenarios for principal component analysis.

Different scenarios—PCA analysis:	
Kept constant	Varied
same mean degree $\hat{d}$ and *δ*	3 network types
skewed-clustered static network	$\hat{d}$ and *β*
skewed-clustered network of same $\hat{d}$, *δ*, *β*	static and 3 turnover rates

$\hat{d}$: mean degree, *δ*: turnover rate, *β*: infection rate

**Table 3:** Summary of the simulation scenarios used for automatic classification.

Different scenarios—automatic classification:	
Predicted variable	Training set
network type	3 networks
*β*	different networks separately
*β*	mis-specified network
*δ*	3 networks
network type in 90 HIV clades/in different fractions of NL tips	3 networks
*β* in 90 HIV clades/in different fractions of NL tips	skewed-clustered network, different *β*

$\hat{d}$: mean degree, *δ*: turnover rate, *β*: infection rate

## Results

The network structure and dynamics both affect features of phylogenetic trees of pathogens spreading on the networks. However, the effects are modulated by the transmission rate and the turnover rate. These relationships are sufficiently strong as to disrupt the signal of the network type in the pathogen phylogeny. A summary of results for the different network structures is given in in the discussion and the trees are given in supporting information.

### Phylogenetic tree features can reveal network structure

**Fig 2:**
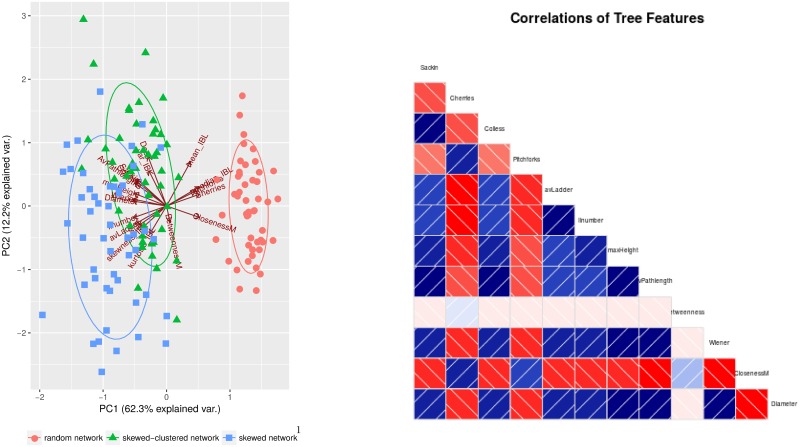
Tree features of the simulated phylogenies. Left: PCA plot of tree features from phylogenetic trees simulated on different networks: random (Erdős-Renyi), skewed and skewed-clustered. Each contact network has mean degree *n* = 5, and all simulated trees have 500 tips. Parameters: infection rate *β* = 0.05, population turnover = 0.03. Right: correlations between tree features, here most features are clearly correlated (blue) or anti-correlated (red). Simulated trees to figure (a) are found in supporting information.

Fig 2 shows a principal component analysis based on phylogenetic trees simulated on dynamic networks with three different topologies. Phylogenies from the Erdős-Renyi network differ strongly from the two others. This holds even for relatively small trees (100 tips), whereas for clustered and unclustered networks, the discrimination improves with the size of tree (up to 250 tips). The same results hold for a wide range of infection rates (*β* = 0.025 to *β* = 0.2) and higher turnover rates (*δ* = 0.1). Overall, the discrimination between networks improves with tree size. The distinction between trees from different underlying networks improves if additional features are used that take into account the lengths of edges. Skewed and skewed-clustered network have a lower number of small substructures (cherries and pitchforks), and a higher value for all imbalance measures. Most network measures (except betweenness) are also positively correlated with imbalance measures.

The network structures become more distinct with a higher rate of infection per contact and with a higher rate of turnover (eg *β* = 0.2, *δ* = 0.1), and in particular the numbers of cherries and the path lengths become more distinct as these parameters increase. Differences in the path lengths and the imbalance between the networks are also more pronounced with higher *β* and *δ*. In contrast, however, there are a few features for which differences are more pronounced at low infection rates (including the ‘ILnumbers’ and the Wiener index for clustered vs unclustered networks). In other words, given fixed values of the transmission and turnover rates, it is possible to separate, and estimate, the underlying network structure based on phylogenetic tree features, for example by discriminant analysis, classification methods, or by Approximate Bayesian Computation.

However, the details—which phylogenetic features point to which kinds of networks—are specific to the transmission and turnover rates, and mis-estimation seems likely if these are mis-specified. Furthermore, for some choices of parameters, the networks are no longer well-separated in the PCA analysis; for example, if *β* = 0.05 and *δ* = 0.1 (so *β* < *δ*), the clustered network overlaps with the random network, whereas if *β* > *δ*, they do not overlap, but the two skewed networks (clustered and unclustered) begin to overlap.

### Features of phylogenies depend on transmission rate and average degree

**Fig 3:**
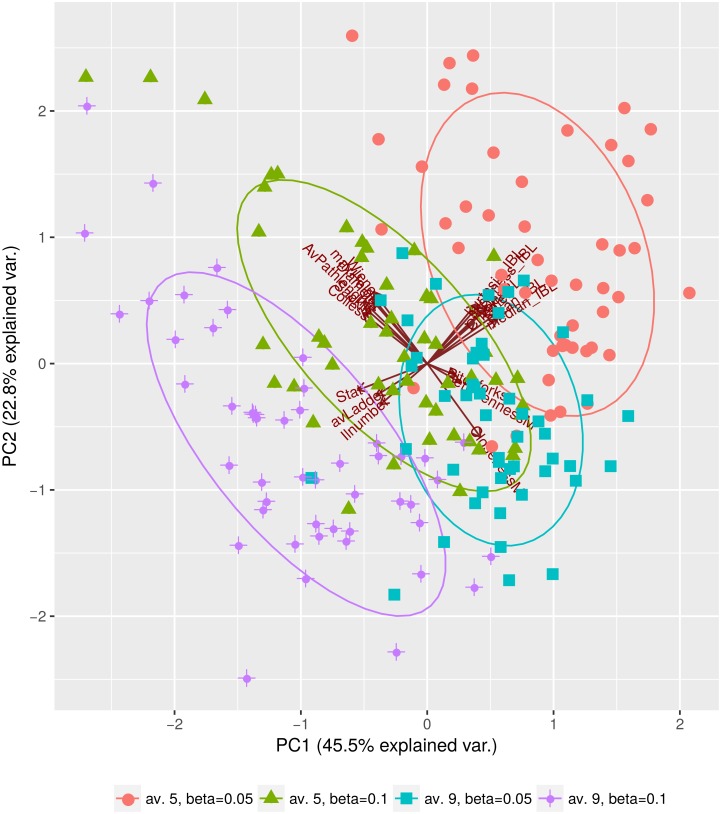
Phylogenies simulated on time-integrated static skewed-clustered networks. We compare trees from outbreaks on networks with mean degrees $\hat{d}=5$ and $\hat{d}=9$ for infection rates *β* = 0.05 and *β* = 0.1. All trees have 500 tips. Simulated trees to this figure are found in S2 File.

When infection rate per contact *β* increases, so does the variance of tree features, and the following tree features increase on average: Colless index, Sackin index, IL numbers (nodes with single tip child), average ladder size, maximum height, average pathlength, Wiener index and diameter. The number of cherries, pitchforks and maximal closeness decrease with increasing infection rate, as shown in Fig 3 for the skewed-clustered network.

**Fig 4:**
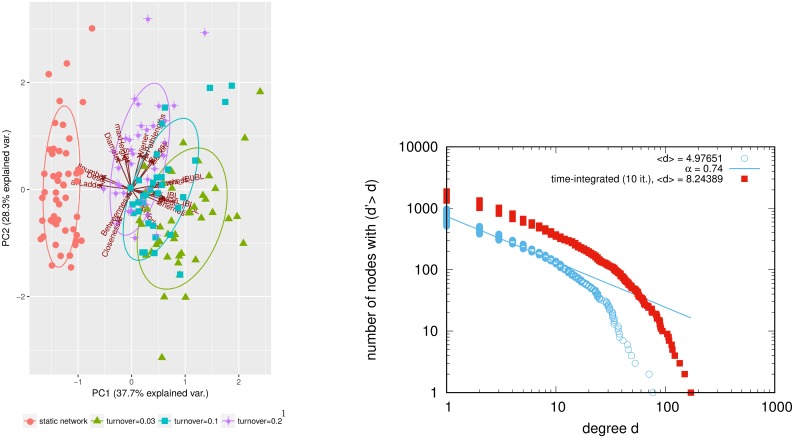
Comparison static vs. dynamic network. Left: PCA plot of tree features for trees from time-integrated and dynamic skewed-clustered networks (*β* = 0.1), mean degree 〈*n*〉 = 5, number of nodes *N* = 1000. red: time-integrated network. Right: counter-cumulative degree distribution on log-log scale of time-integrated and dynamic network.

The same features increase as the mean degree increases (red and green vs. turquoise and purple in Fig 3), which is expected, as both increasing *β* (infection rate per contact) and increasing the number of contacts increase the basic reproduction number $R_{0}=\beta \bar{d}\tau$ (*τ* being the duration of infection and $\bar{d}$ the median degree) of an outbreak. The phylogenies from the four outbreak hypotheses in Fig 3 may therefore correspond to different pathogens or to a pathogen in rather different epidemiological settings, as in these scenarios *R*_0_ values may differ substantially. However, the tree features that discriminate these scenarios are also affected by the nature of the contact network (Fig 1) and by the turnover rate (Fig 4). This comparison highlights that the network type and turnover are likely to affect estimation of the mean degree and the infection rate from phylogenetic trees.

Simulated trees to figure 4 are found in S3 File.

### Network dynamics affect phylogenetic tree features

Fig 4 shows a PCA of phylogeny features derived from skewed-clustered networks with same mean degree but different turnover rates (i.e. rates at which people enter and exit the system), and from a time-integrated static network of same mean degree $\hat{d}$. Higher population turnover of the network increases the following features of the simulated phylogenetic trees: Sackin index, Colless index, average ladder sizes, IL number, maximum height, average pathlengths, diameter, Wiener index, and betweenness, and decreases the number of cherries and pitchforks as well as maximum closeness.

Higher turnover gives similar results to a higher mean degree or a higher infection rate (see Fig 3). The static time-integrated network has no turnover, but contacts have a longer duration, presenting the opportunity to transmit comparably to a dynamic network with much higher turnover than the one used for the time integration. In dynamic networks, links get rewired often and therefore many opportunities for transmission exist. The static network has higher mean degree as the temporally existing links are accumulated (see Fig 4). Instead of resembling those from very low turnover, the phylogenies from static networks have therefore features similar to those from networks with very high turnover. This effect holds for different infection rates *β*, but the higher the infection rate, the more the phylogenies from a time-integrated network differ from those from networks with low turnover.

Results for varying infection rate, mean degree, turnover and time-integration are qualitatively the same for the skewed-clustered and skewed-unclustered network, but since the unclustered network has shorter average pathlength than the clustered network of same mean degree, the effects are more pronounced.

Imbalance measures are always anticorrelated with the counts of small substructures (pitchforks and cherries). The fact that network skewness increases tree imbalance (and decreases substructures) could be due to the fact that high heterogeneity in the network degree is passed on to high heterogeneity in the number of secondary infection, resulting in an imbalanced tree (measured e.g. by Sackin and Colless index). On the other hand, increased network clustering may have the opposite effect, as it results in fewer nodes being connected to hubs in the network, which may cause the infection tree and resulting phylogenetic tree to be more balanced and to exhibit more pitchforks and cherries. However, an imbalanced phylogenetic tree could in principle also result from long chains of person-to-person transmission, in which each individual infects exactly one other: imbalanced trees do not necessarily require heterogeneous contact numbers or heterogeneous numbers of secondary infections.

### Classification of networks and parameters from phylogeny features

For simulations with distributed values for *β*, *δ* and mean degree of the network, we calculated all of our features of phylogenetic trees and used these to train classifiers, which we then tested. We used K nearest neighbours (KNN) [58] which classifies an object based the the class of the majority of its nearest neighbours, and random forests [59] which use decision trees to classify the test data. We simulated 1549 phylogenetic trees on the three types of networks, with random uniformly distributed values of the turnover and transmission rate parameters (both in [0.05, 0.15]) and mean degrees (in [4, 9]). We trained classifiers on 1040 instances to classify from which type of network a phylogeny was derived. We compute the mean and standard deviation of the accuracy using 10-fold cross-validation. The classification is successful in the sense that it is possible to classify the dynamic network type based on the phylogenetic features, given a range of transmission parameters and turnover rates in the training data. Table 4 lists the results when we choose the key parameters *β* (transmission rate), mean degree and turnover *δ* uniformly at random over the specified ranges. Both classifiers predict the network type with high accuracy, using the phylogenetic features. This means that even with the additional complications of dynamic networks and unknown underlying parameters, phylogenetic trees encode information about the nature of the network.

**Table 4:** Prediction accuracies (correctly predicted/all predictions).

Predicted value	Accuracy (knn)	Accuracy (random forest)	Size of training set	Size of test set
All three networks, range of mean degree and turnover				
network	0.88 ± 0.02	0.92 ± 0.01	1084 (3 networks)	465 (3 networks)
*β*	0.40 ± 0.01	0.47 ± 0.03	1084 (3 networks)	465 (3 networks)
Correctly specified network, range of mean degree and turnover				
*β*	0.38 ± 0.04	0.43 ± 0.05	261 (skewed-clustered)	113 (skewed-clustered)
*β*	0.39 ± 0.04	0.55 ± 0.04	262 (skewed)	113 (skewed)
*β*	0.39 ± 0.03	0.44 ± 0.03	560 (random)	240 (random)
Mis-specified network, range of mean degree and turnover				
*β*	0.30 ± 0.01	0.37 ± 0.01	800 (random)	374 (skewed-clustered)
*β*	0.34 ± 0.01	0.39 ± 0.01	375 (skewed)	374 (skewed-clustered)
*δ*	0.36 ± 0.03	0.45 ± 0.03	1084 (3 networks)	465 (3 networks)

Predictions of network type, infection rate *β* and turnover rate *δ*. Values are mean and standard deviation of 10-fold cross-validation. For this, *β* (and *δ* respectively) is grouped into bins of width 0.01. *β* is considered to be classified correctly if it is classified into the correct or in neighbouring bins (i.e. in a range of 0.03). For the simulations, infection rate *β* and turnover *δ* are both distributed uniformly at random in [0.05, 0.15], and mean degree $\hat{d}$ between [4, 9] respectively. Simulated trees to this table are found in S4 File.

We also asked how varying the underlying (and in general unknown) dynamic contact network would affect estimation of the transmission parameter *β* (also in Tables 4 and 5). Estimation of *β* is much worse than estimation of the network, and strongly depends on the assumed network. The performance is best for random forests with either all three networks present in the data (accuracy 0.47) or with a single, correctly-specified, skewed or random network used to train the model (accuracy 0.55, 0.44 respectively). Mis-specification of the network worsens predictions.

**Table 5:** Prediction accuracies (correctly predicted/all predictions).

Predicted value	Accuracy (knn)	Accuracy (random forest)	Size of training set	Size of test set
All three networks, constant mean degree and turnover				
network	0.92 ± 0.02	0.94 ± 0.01	599 (3 networks)	258 (3 networks)
*β*	0.52 ± 0.04	0.69 ± 0.02	599 (3 networks)	258 (3 networks)
Correctly specified network, constant mean degree and turnover				
*β*	0.37 ± 0.04	0.39 ± 0.05	105 (skewed-clustered)	45 (skewed-clustered)
*β*	0.39 ± 0.05	0.67 ± 0.03	227 (skewed)	99 (skewed)
*β*	0.7 ± 0.04	0.82 ± 0.03	267 (random)	116 (random)
Mis-specified network, constant mean degree and turnover				
*β*	0.32 ± 0.03	0.29 ± 0.01	382 (random)	150 (skewed-clustered)
*β*	0.25 ± 0.02	0.23 ± 0.02	325 (skewed)	150 (sk-cl.)

Predictions of network type and infection rate *β*. For this, we simulated outbreaks on dynamic networks with varying *β*. *β* is grouped into bins of width 0.005 (while for simulations *β* has been distributed in [0.05, 0.1]. We assume *β* to be correctly classified if it fits within the same or the neighbouring bins for *β*, i.e. in 3 of the 11 possible bins, so random allocation into bins would result in an an accuracy of 0.27. For network prediction, random allocation would give an accuracy of 0.33. The results show mean and standard deviation of 10-fold cross-validation. Results show that if the classifier is trained on the wrong network, the prediction accuracy is much lower. Results also show that in comparison to the results in Table 4, higher accuracy is obtained for the prediction on skewed and random network, since turnover and mean degree are fixed, although the range of *β* is only 0.05. Turnover (*δ* = 0.1) and mean degree ($\hat{d}=5$) are fixed throughout the tests listed here. Simulated trees to this table are found in S5 File.

Discrimination between skewed and skewed-clustered networks remains difficult, as these networks are quite similar. The difference between skewed and random networks is more pronounced (as also seen in the PCA analysis in Fig 2). In that sense our results are similar to the results in [60–62], who successfully predicted contact rates with Approximate Bayesian Computation (ABC) on static networks, where the phylogenetic trees separate well in a PCA plot of extracted tree measures.

Given the poor ability to predict *β* when the mean degree and turnover are randomly sampled, we explored whether keeping these parameters fixed would improve the estimation: if we knew these parameters and had pathogen phylogenies, would we then be able to estimate the transmission rate in the context of dynamic networks? Here, the accuracy is only good in the case of the random network (0.7, 0.82 for KNN, random forests respectively). Random forests give consistently slightly higher accuracy, with an accuracy over 0.5 where (1) all three networks (skewed, skewed-clustered an random) were present in the training data, or (2) the model was trained on the skewed or random networks. If the network is mis-specified or skewed, neither approach is able to predict *β*. We suggest that this may have adverse consequences for analyses using static or other assumed network models in phylodynamics; these may draw erroneous conclusions about the rate of transmission or other parameters due to mis-specification of the underlying network.

### Classification of HIV data

We trained classifiers on phylogenetic trees simulated with different network hypotheses, in order to predict the network type for HIV clades from sequences of patients in the Dutch ATHENA cohort and from sequences of the Los Alamos Sequence database [55]. The Dutch sequences predominantly capture the Dutch national HIV epidemic (cite Bezemer PLoS Med), whereas the sequences in the Los Alamos database are from cases worldwide and capture many diverse HIV epidemics. Our network predictions are consistent with this: the higher the fraction of tips from the Netherlands, the more HIV trees are predicted to arise from skewed or skewed-clustered networks, rather than random (see Table 6); this signal is consistent in the K-nearest neighbour and random forest classification.

**Table 6:** Classification of HIV trees into trees from 3 simulated networks.

trained on: trees from all three networks, mean deg. 〈*n*〉 = 5 and turnover *δ* = 0.1				
HIV test clades	KNN		Random forest	
	s/sc network	r network	s/sc network	r network
all 90	0.49	0.51	0.43	0.57
with >50% NL-tips	0.81	0.19	0.87	0.13
with >70% NL-tips	1	0	1	0
with <30% NL-tips	0.38	0.62	0.30	0.70

Classification of HIV trees with different fractions of tips from the Netherlands (NL). Ratios of HIV trees classified into a network type network type of HIV trees. For this, we simulated 857 trees on three networks truncated to 100 tips, trained KNN and random forest classifiers on them, and tested them on HIV trees and subsets that have a certain fraction of tips from the Dutch dataset. For these results, tree features were calculated with all branchlengths being set to 1, to make simulated trees and HIV trees comparable. Simulated trees and anonymized HIV trees to this table are found in supporting information.

We also trained the classifiers on simulated trees from a skewed-clustered network with two different infection rates (*β* = 0.05 and *β* = 0.2), in order to predict the infection rate for the HIV trees (see Table (7). We did the latter both with trees from static networks and dynamic networks with turnover rate *δ* = 0.1. For the static network, roughly two thirds of the HIV trees are predicted to have infection rate *β* = 0.05 and one third *β* = 0.2. In contrast, all of the HIV trees are predicted to have the higher infection rate of *β* = 0.2 on the dynamic network.

**Table 7:** Classification of HIV trees into simulated trees from outbreaks with different *β*.

HIV test clades	KNN		Random forest	
	*β* = 0.05	*β* = 0.2	*β* = 0.05	*β* = 0.2
trained on: trees from **static** skewed-clustered networks				
all 90	0.63	0.37	0.66	0.34
with >50% NL-tips	0.23	0.77	0.08	0.92
with >70% NL-tips	0.12	0.88	0	1
with <30% NL-tips	0.71	0.29	0.75	0.25
trained on: trees from **dynamic** skewed-clustered networks with *δ* = 0.1				
all 90 clades	0	1	0	1
with >50% NL-tips	0	1	0	1
with >70% NL-tips	0	1	0	1
with <30% NL-tips	0	1	0	1

We classified the HIV trees into trees from a skewed-clustered network with different infection rates. This has been done for a static network and for a dynamic network (*δ* = 0.1). We predicted the parameters for 90 HIV trees (of which 13 had 50% of tips from the Netherlands, and 7 more than 70%). Sizes of the training sets for the classifiers are 400 and 244. As in Table 6, branchlengths of simulated trees were not used. Simulated trees and anonymized HIV trees to this table are found in S6 File.

It is not surprising that more HIV trees were predicted to have the higher infection rate *β* = 0.2 when the classifiers were trained on the dynamic network. On dynamic networks, not all links are present at any moment, which slows down the outbreak. A higher infection rate could compensate to attain the same R0. This result was very robust even when fewer tree features were used to train the classifier. However, if only imbalance measures were used, a low fraction of HIV trees were predicted to have *β* = 0.05 by dynamic-network-based classifiers. This suggests that using a variety of tree features is important for specification of network parameters from phylogenies.

We have also listed separate predictions for clades in which more than 50% or 70% of the tips are from the ATHENA dataset; these are geographically linked, may include more recent transmission and are likely to have a higher sampling density than background clades from the Los Alamos database. Compared to the whole set of 90 HIV clades, these clades are more likely to be classified to have come from a skewed (clustered) network and to have a high transmission rate (*β* = 0.2). However, the certainty on this prediction depends on the underlying network assumption, with classifiers trained on dynamic models showing a completely consistent set of predictions while those trained on static models leave considerable variation (Table 7). In contrast, clades with fewer Dutch sequences were classified predominantly to have a lower transmission rate if classifiers were trained using static networks, but a higher transmission rate using dynamic networks. The fact that the results differ considerably depending on the underlying network assumption indicates that a mis-specified network, via an incorrect turnover rate or indeed the assumption of a static network, can have a strong effect on predicted transmission rates.

## Discussion

We used models of different human host contact networks to simulate outbreaks of pathogens, and convert the infection trees into phylogenetic trees. We showed that it is possible to discriminate with tree statistics between different contact network hypotheses, different turnover rates, different mean degrees and different infection rates. Table 8 sumarizes the network effect on tree statistics. The underlying contact network hypothesis (random, skewed or skewed-clustered) is clearly identifiable in statistics of the simulated phylogenetic trees, if *β* and *δ* are the same. This indicates that simple networks such as the Erdős-Renyi model are likely to be unsuitable models for human host contact networks where there is evidence for a skewed degree distribution and clustering.

**Table 8:** Summary of the direction of change of features when the network parameters (left column) are varied in the indicated direction.

network parameter	imbalance measures	cherries/pitchforks	mean internal branch length	IL numbers/LadderSize
turnover *δ* ↑	(slightly)↑	↓	↓	↑
skewed degree dist ↑	↑	↓	↓	↑
clustering ↑	↓	↑	↑	↓
infection rate *β* ↑	(slightly) ↑/ –	(slightly) ↓	↓	↑
mean degree $\hat{d}$ ↑	(slightly)↓	(slightly)↓	↓	↑

Nevertheless, in our simulations, phylogenies from skewed-clustered networks are slightly more similar to those from random networks than those from unclustered networks of the same degree distribution. Phylogenetic trees from outbreaks on the same static network, but with different infection rates or different mean degrees, can be distinguished clearly in PCA plots. This result holds also on dynamic networks, and suggests, in keeping with previous work, that phylogenetic tree features can be used to estimate epidemiological parameters. However, the relationships between the epidemiological parameters, networks and phylogenetic trees are complex. We tested the strength of some of these relationships using supervised learning methods, and found that both network mis-specification and variability in other parameters (modelling uncertainty about the values of these parameters) have a strong impact on the ability to estimate the transmission parameter. Our results indicate that consistent network mis-specification and parameter uncertainty may have an adverse impact on phylodynamic studies estimating parameters from data.

Population turnover in dynamic networks has a measurable effect on pathogen phylogenies; phylogenetic tree features can discriminate between different turnover rates at which the underlying network is evolving. Overall, the higher the turnover, the higher the imbalance measures and the lower counts of small substructures. No single feature captures the differences between contact network hypotheses entirely, and a combination of many different features yields the best visual separation between the groups in a PCA plot. Features that take into account the branch length of the phylogenetic trees improve the separation slightly. Very different patterns are obtained from a static time-integrated network as compared to dynamic networks, on which transmission happens slower. This suggests that in the phylodynamic setting, static networks are a poor approximation for dynamic networks, highlighting the need for dynamic network models. This also highlights the need for investigating turnover and dynamics in empirical networks to obtain the data necessary to develop dynamic models. We illustrated this result by predicting the infection rate *β* of HIV trees, and showed that the predictions strongly underestimate *β* if a static network is used instead of a dynamic one. Comparison to HIV data also showed that clades with tips predominantly from the Dutch sequence dataset with high sampling fraction of infected individuals are more likely to be predicted to have come from a skewed or skewed-clustered network than those with tips mainly from the even sparser sampled Los Alamos database.

Although the dynamic skewed-clustered network is likely to be a more realistic approximation to real networks than static or unclustered networks, it still might not be as clustered as a given real contact network. The details of the relevant network for a study of real data will depend on the pathogen and also on the nature of the community in which that pathogen is being studied. The dynamic models we have used here are still relatively simple and tractable, and real networks are likely to be even more heterogeneous.

## Supporting information

S1 File Compressed .RData file of the trees for Fig 2a in phylo format. (ZIP)

S2 File Compressed .RData file of the trees for Fig 3 in phylo format. (ZIP)

S3 File Compressed .RData file of the trees for Fig 4 in phylo format. (ZIP)

S4 File Compressed .RData file of the trees for Table 4 in phylo format. (ZIP)

S5 File Compressed .RData file of the trees for Table 5 in phylo format. (ZIP)

S6 File Compressed .RData file of the trees for Table 6 in phylo format. It contains HIV trees which have anonymized tips.
(ZIP)
